# Nitrogen Assimilation in the Freshwater Amphipod *Gammarus fossarum* is Dominated by Animal‐Derived Resources

**DOI:** 10.1002/ece3.73556

**Published:** 2026-04-21

**Authors:** Katharina Amann, Rebecca Hoess, Karl Auerswald, Juergen Geist

**Affiliations:** ^1^ Aquatic Systems Biology Unit, TUM School of Life Sciences Technical University of Munich Freising Germany

**Keywords:** aquatic food web, functional feeding type, litter decomposition, δ^15^N

## Abstract

Understanding how consumers assimilate nutrients from different resources is central to predicting nutrient cycling in ecosystems. Amphipods of the genus *Gammarus* are abundant detritivorous macroinvertebrates in European headwater streams, yet the relative contribution of plant‐ and animal‐derived nitrogen (N) to their biomass remains poorly quantified. We conducted a 14‐day laboratory experiment with the amphipod *Gammarus fossarum* using a stable isotope (δ^15^N) tracer approach. Individuals were supplied with either black alder leaves ( *Alnus glutinosa* ) or highly ^15^N‐enriched hay to trace nitrogen assimilation and estimate tissue turnover rates. Despite large differences in isotopic values and nitrogen availability between plant resources, assimilation of plant‐derived nitrogen was minimal. Instead, isotopic values indicated that most assimilated nitrogen originated from animal material, most likely through cannibalism. The estimated turnover rate constant was 0.10, corresponding to a nitrogen half‐life of 6.7 days, with approximately 76% of metabolically active nitrogen replaced during the experiment. Our results show that nitrogen assimilation in *G. fossarum* can be dominated by animal‐derived resources even when plant detritus is abundant. This highlights the importance of trophic flexibility in detritivorous macroinvertebrates and suggests that animal‐derived nutrients may play a larger role in detritus‐based stream food webs than commonly assumed.

## Graphical abstract

Based on a systematic feeding experiment with isotopically labeled feed, we challenge the current view on feeding patterns and nutrient processing in gammarids, with opportunistic cannibalism playing a more important role for N‐uptake than the incorporation of N from different plant materials.

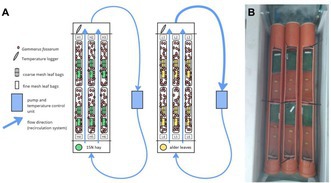

## 1 Introduction

Freshwater ecosystems play a central role in global carbon and nutrient cycling by mediating the transformation of organic matter and transferring energy across trophic levels. Terrestrial organic matter, including leaves, grasses, and other detrital inputs, often constitutes a major source of nutrients for aquatic food webs, particularly in headwater streams and small tributaries (Tank et al. 2010; Wallace and Webster 1996; Allen et al. 2024). Once transported into the stream, coarse particulate organic matter is colonized by microbial communities and consumed by detritivorous invertebrates, thereby linking terrestrial production to aquatic consumers and higher trophic levels (Hieber Ruiz and Gessner 2002). Macroinvertebrates classified as shredders, such as many gammarid amphipods, facilitate microbial decomposition by fragmenting coarse detritus into finer particles and accelerating nutrient turnover, which in turn enhances heterotrophic energy flow and ecosystem functioning (Navel et al. 2011).

The availability and quality of terrestrial detritus are shaped by both natural riparian vegetation and anthropogenic land‐use practices. In many streams, the quantity, timing, and quality of inputs from leaf litter and other detrital material have been altered due to changes in forestry practices, agricultural management, and climate‐driven changes in vegetation dynamics (Wild and Geist 2025; Lyons et al. 2000). Such changes affect the nutritional composition of detritus, including carbon‐to‐nitrogen (C:N) ratios, lignin content, and microbial colonization, which together influence the palatability and digestibility of plant material for detritivores (Bloor 2011; Hladyz et al. 2011; Menninger and Palmer 2007). While certain leaf types, such as those of *Alnus glutinosa* , are traditionally considered high‐quality detritus due to low C:N ratios and low lignin content, freshly cut herbaceous material like hay can offer even higher digestibility, albeit with variable nitrogen content. Understanding how detritivores respond to these changes in detrital quality is crucial for predicting nutrient cycling and energy flow in freshwater systems and for understanding their functional ecology.

Among shredders, the amphipod *Gammarus fossarum* is widely distributed in Central European headwater streams and frequently dominates macroinvertebrate communities (Weiss and Leese 2016). Traditionally classified as a “shredder‐detritivore,” *G. fossarum* is assumed to process coarse plant material, thereby facilitating microbial decomposition and making nutrients available to other consumers (Wallace and Webster 1996; Cereghetti et al. 2025). By enhancing the turnover of terrestrial detritus, gammarids link basal resources to higher trophic levels and play a pivotal role in sustaining the stability and productivity of headwater ecosystems (Yang et al. 2024). However, increasing evidence indicates that gammarids can also be generalist consumers whose diets also include animal tissue, including conspecifics (Dick 1992, 1995; Kelly et al. 2002a; Alther et al. 2023). Laboratory studies and field observations show that *Gammarus* species readily consume weakly mobile invertebrates, exhibit intraguild predation, and can engage in cannibalism, particularly under certain ecological conditions (MacNeil et al. 1997; Felten et al. 2008; Georgievová et al. 2020; Syrovátka et al. 2020).

Despite the prevalence of animal material in their diet, the extent to which gammarids assimilate nitrogen (N) from plant versus animal sources remains poorly understood. While gut content analyses often reveal plant fragments, nutrient assimilation appears to occur predominantly through microbial biofilms and animal prey rather than directly from plant tissue (France 2011; Nelson 2011). The discrepancy between conventional functional classifications (e.g., Moog 1995; Schmidt‐Kloiber and Hering 2015), which assign low predatory feeding scores to *Gammarus*, and empirical evidence showing substantial predatory and cannibalistic feeding, has important implications. Accurate characterization of their feeding ecology is essential for understanding nutrient cycling, predicting community responses to environmental change, and designing ecotoxicological experiments that rely on gammarids as model organisms (Feiner et al. 2015; Götz et al. 2022).

Stable isotope analysis provides a powerful tool to trace the assimilation of different nutrient sources in food webs (Vander Zanden et al. 2015; Eglite et al. 2023). Enrichment of the heavier N isotope (^15^N) occurs predictably along trophic gradients, enabling the quantification of animal‐derived versus plant‐derived N assimilation (Boecklen et al. 2011; Layman et al. 2012). Experimental labeling with ^15^N allows for mechanistic insights into nutrient uptake and turnover, including the estimation of metabolic N replacement rates and the relative contribution of cannibalism to overall diet. Such approaches are particularly valuable for small‐bodied macroinvertebrates like *G. fossarum*, where direct observations of feeding behavior may not fully capture dietary plasticity.

In this study, we conducted a controlled laboratory experiment using ^15^N‐labeled hay and alder leaves to quantify the relative contributions of plant‐derived and animal‐derived N in *G. fossarum*. We aimed to (i) assess the extent of plant N assimilation, (ii) test potential preferences for different plant resources and (iii) quantify whole‐body N turnover in *G. fossarum*. Based on previous literature, we hypothesized that:

*G. fossarum would primarily assimilate plant‐derived N as a shredder–detritivore*.

*G. fossarum would preferentially consume softer, more digestible hay compared with alder leaves*.

*Nitrogen incorporated from plant resources would be progressively integrated into gammarid biomass, allowing estimation of whole‐body N turnover*.

By explicitly considering cannibalism and its potential contribution to N assimilation in our data analysis, this study aims to improve the mechanistic understanding of how trophic plasticity shapes nutrient processing in freshwater detritivore communities.

Overall, our approach integrates contemporary ecological understanding of land‐use impacts, detrital resource quality, and omnivorous feeding behavior, moving beyond traditional classifications of gammarids as purely shredders. This perspective aligns with the growing emphasis in functional ecology on species traits, trophic plasticity, and ecosystem‐level nutrient fluxes, offering a more nuanced and realistic view of food web dynamics in headwater streams.

## 2 Material and Methods

### 2.1 Study Species

*Gammarus fossarum* was used as a model freshwater detritivore due to its key role in leaf litter processing in European streams. Individuals were collected from the Otterbach stream, a siliceous low mountain stream in Bavaria, Germany (mean discharge 0.798 m^3^ s^−1^). The sampled reach was located in an open agricultural landscape without riparian tree cover. To obtain individuals of similar size, the specimens were carefully flushed through two buckets with perforated bases (2–4 mm). *G. fossarum* individuals were starved for 24 h prior to the start of the experiment to ensure empty guts. The length of each individual was measured from the 1st thoracic segment to the base of the uropod before the experiment using a stereomicroscope (OLYMPUS, SZX10, Olympus, Tokyo, Japan) and a measurement tool (CellSens Entry 2.2, Olympus, Tokyo, Japan). Individual wet weights were determined before the experiment using a precision scale with an accuracy of 0.01 mg (Sartorius R200D, Sartorius GmbH, Göttingen, Germany). To determine baseline dry mass and ^15^N values, 24 individuals were oven‐dried at 60°C immediately after the initial measurements.

### 2.2 Study Design

**FIGURE 1:**
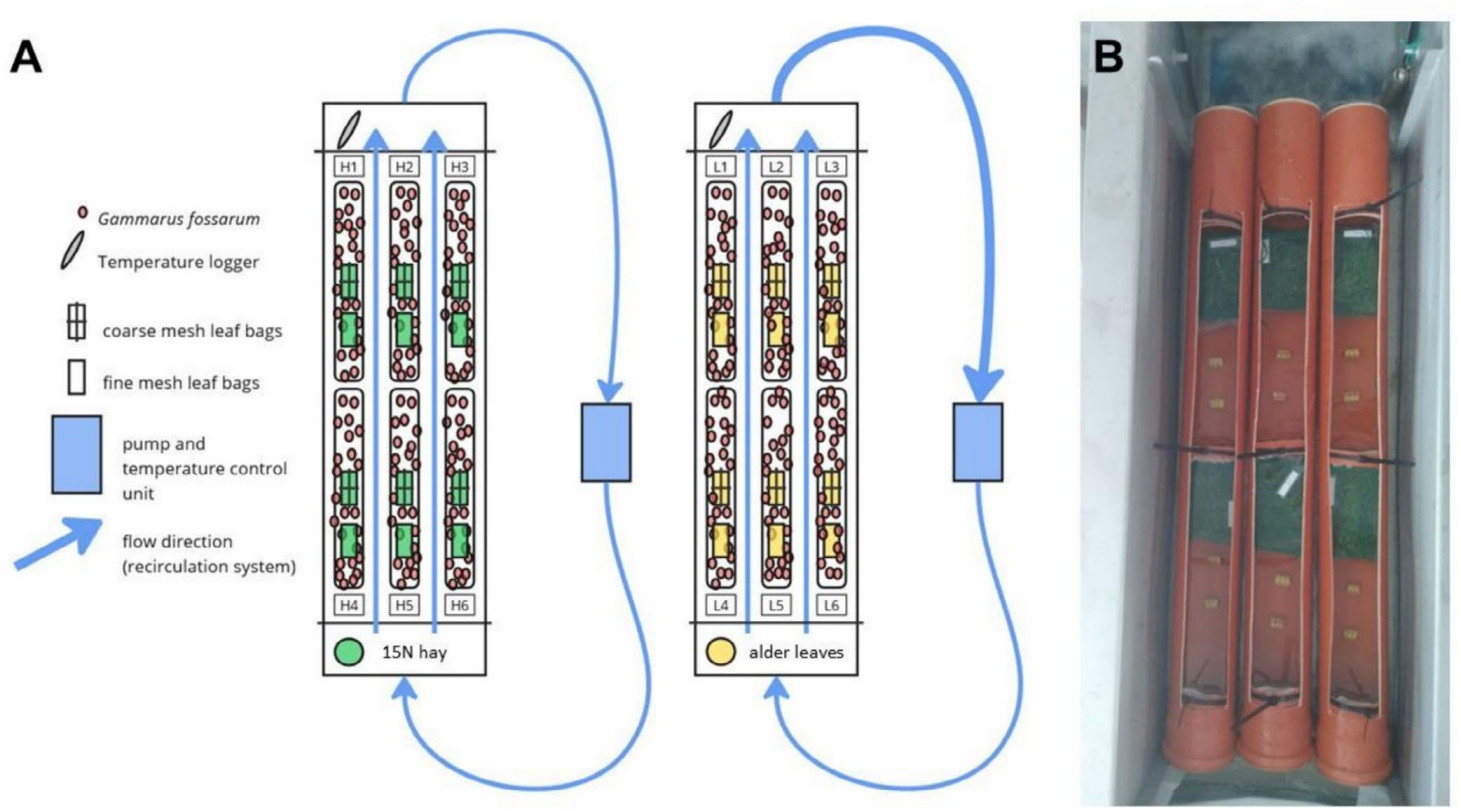
(A) Overview of the experimental setup with two recirculation flumes, equipped with temperature loggers, and separated into six compartments for the exposure of either hay (compartment H1–H6) or alder (compartment L1 – L6) litter bags. (B) Photo of the experimental setup.

To investigate N assimilation and turnover in *G. fossarum*, we conducted a controlled laboratory flume experiment (Figure 1). The experiment was carried out in October 2023 and lasted 14 days. The day on which gammarids were introduced into the experimental compartments was defined as Day 0. Each flume had a volume of 44.4 L and was equipped with a flow circulation system in which temperature (14°C) and flow velocity (approximately 0.11 m/s) were regulated. Three plastic tubes (inner diameter 110 mm, length 1150 mm) with rectangular openings measuring 100 × 1050 mm cut out at the top were placed onto the flume base. The water level reached half of the tube diameter. Each tube was separated into two replicate compartments (volume 3250 cm^3^) by gauze nets (0.5 mm mesh size), which were also used to cover the open ends of the tubes. The flumes were filled with water from the Otterbach and circulated for 2 days before the start of the experiment to ensure similar temperatures and flow velocities within each compartment.

To test H1 and H2, two different types of plant material were prepared as a food resource. Alder leaves ( *Alnus glutinosa* ) were collected from the banks of the Otterbach stream and are commonly used as food for gammarids in laboratory studies (Hieber Ruiz and Gessner 2002). Only freshly fallen leaves were collected to minimize fungal infection. Hay from a semi‐natural grassland in the Otterbach catchment was used as an isotopically labeled plant resource. The grassland vegetation was dominated by *Dactylis glomerata, Alopecurus pratensis*, and *Plantago lanceolata* (Martínez‐Cuesta et al. 2024). The hay had previously been labeled with ^15^N through fertilization with ^15^N‐enriched fertilizer (Floßmann et al. 2025), resulting in strongly elevated δ^15^ values. The strong labeling enabled tracing of ^15^N accumulation within the study organism over time to characterize body turnover rates and thus test H3.

Plant material was oven dried at 60°C for 72 h, then two types of litter bags were filled with 4.1 ± 0.2 g of one of the food resources: coarse litter bags allowed direct access by the gammarids (coarse mesh, 4 mm; HDPE), and fine litter bags (fine mesh, 0.15 mm; polyamide) excluded access. After system equilibration, one coarse and one fine litter bag filled with either alder leaf litter (“alder treatment”) or ^15^N labeled hay (“hay treatment”) were placed in each compartment, with one flume equipped only with alder and the other only with hay bags to avoid contamination of the alder flume with ^15^N‐rich leachate from the hay. Each compartment was supplied with 34 individuals of *G. fossarum*, allowing for a weekly removal of 12 individuals for subsequent analyses and providing an additional buffer of 10 individuals to account for potential mortality.

After 7 days, 12 gammarids were removed from each compartment. Individuals were starved for 24 h to allow gut clearance, measured under a stereomicroscope, dried at 60°C for 24 h, and weighed. After 14 days, all remaining gammarids and litter bags were removed from the flumes. Individuals were again starved for 24 h, measured, dried, and weighed. In some compartments, fewer individuals were recovered than initially introduced. As no dead individuals or exoskeleton remains were observed in the flumes, these losses were attributed to cannibalism.

### 2.3 Weight Loss of Litter

Litter mass loss was determined by weighing the litter bags before and after the experiment after drying at 60°C for 72 h. After drying, the biomass was weighed using a precision balance. The organic content of the plant material was determined by loss‐on‐ignition, combusting samples at 500°C for 4 h in a muffle furnace.

### 2.4 Stable Isotope Analysis

Prior to isotope analysis, all dried *G. fossarum* individuals were crushed individually using a ceramic mortar and 0.6–0.9 mg of crushed biomass was packed into small tin capsules (5 × 9 mm). Six biomass samples were taken on Day 0 and Day 14 to analyze the C:N ratio and the δ^15^N value of alder leaves and hay biomass. Both plant types were first homogenized and pulverized using a ball mill (Retsch GmbH, Haan, Germany). All dry mass was analyzed except alder main veins. To calculate total N concentrations and the ^15^N:^14^N isotope ratio, the samples were analyzed using an elemental analyzer (Flash EA, Thermo Scientific, Waltham, USA) coupled to an isotope ratio mass spectrometer (Delta PlusXP, Thermo Scientific, Waltham, MA, USA; Schreiber et al. 2023). Each sample was calibrated against a set of certified laboratory standards (IVA Analysetechnik, Meerbusch, Germany). A low‐organic soil standard was run in four different amounts to enable correction of amount‐dependent effects. After every eleventh sample, an L‐glutamic acid standard was run as a drift standard. A sorghum flour standard was used as a long‐term quality control. The precision of repeats (SD) was less than 0.23‰ for the laboratory standards. In addition, an alfalfa standard was run to calibrate the N content. Isotope data are presented in δ notation as:

(1) $$\delta^{15}N=R_{\text{sample}}/R_{\text{standard}}–1$$

where *R* is the ^15^N:^14^N abundance ratio and *standard* refers to the International Atomic Energy Agency AIR standard for N.

The δ^13^C values were also measured but are not reported here, as the isotopic spacing between the offered food resources and gammarids was insufficient to provide meaningful information on turnover.

### 2.5 Data Analysis

**Table:** 

Scale of inference	Scale at which the factor of interest is applied	Number of replicates at the appropriate scale
Experimental units	Compartments of flume mesocosms	2 food sources × 6 compartments each

#### 2.5.1 Calculation of N Masses and Losses

N dynamics in the experimental system were quantified by calculating N masses and N losses for both plant material and gammarid biomass. N mass of plant material was calculated as the product of N content and dry mass at Days 0 and 14. N mass of gammarid biomass was calculated from individual dry mass and N content for each sampling date. Changes in δ^15^N values of gammarids over time were used to estimate whole‐body N turnover.

**FIGURE 2:**
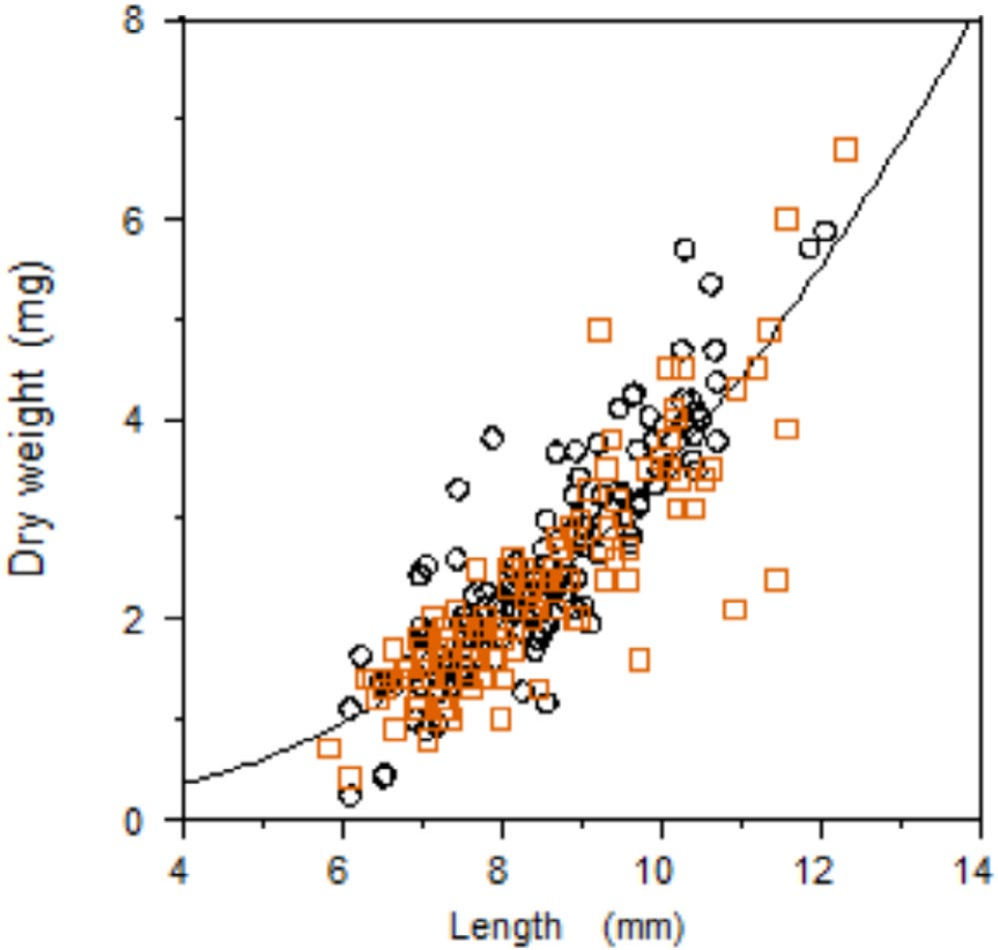
Allometric relation between body length and dry weight of *Gammarus fossarum*. Data were obtained after Day 7 (circles) and Day 14 (squares).

For weight changes and N mass changes of gammarids, allometric relationships between length and dry weight were calculated using power regression on data obtained on Day 7 (*n* = 139) and Day 14 (*n* = 115) and used to calculate the dry weight on Day 0. Non‐linear relationships were comparable for both days, that is, a dummy variable for the group and an interaction term were not significant when the data were combined. The combined data without dummy variable and interaction term yielded the back‐transformed power regression:

(2) $$W=0.01\times L^{2.54}$$

where *W* denotes the dry weight in mg, and *L* denotes the length in mm (*n* = 254; *R* ^2^ = 0.709; Figure 2). Out of 442 required weight data points, five were missing and were estimated using the allometric relation given in Equation (2).

The gammarid N mass was given by the product of the number of individuals, mean dry weight and N content. The number of individuals on Day 14 was lower than on Day 0 because individuals were removed on Day 7 and others were cannibalized. We assumed that the Days 7–14 disappearance rate would also apply between Days 0–7, although cannibalism may have been higher at higher densities, that is, between Days 0–7 compared to Days 7–14. This allowed estimation of the number of individuals that would have been present on Day 14 had individuals not been removed on Day 7. Linear regression was used to assess the decrease in N content of gammarid biomass per compartment over time.

#### 2.5.2 Estimation of Body Turnover

Due to the small mass of gammarids, the N half‐life was expected to be considerably < 14 days (Braun et al. 2013) and the experiment should thus have been long enough to turn over most of the metabolically active body tissues.

Turnover follows an exponential decay function but can be obscured by inert body tissues like the exoskeleton (Auerswald et al. 2010) and by growth (Fry and Arnold 1982; Carleton and Del Rio 2010). Body mass did not change and growth therefore did not require consideration, and a simple decay function was used (Sponheimer et al. 2006; Cerling et al. 2007):

(3) δNt=15δNfinal+(δNinitial−1515δNfinal)×e−r×t15

where *initial* and *final* denote the values at the start of the experiment and after full turnover, respectively. The exponents are the turnover rate constant *r* and the time *t* since feeding the labeled plant material.

The final value, δ^15^ *N*_*final*_, should be equal to the isotopic value of the plant material (δ^15^ *N*_*feed*_) plus the trophic shift, which reflects the enrichment of the heavier isotope at the next higher trophic level. In the case of ^15^N, the trophic shift was assumed to be 3‰ (Post 2002). Thus, the isotopic value of the digested food resource is given by δ^15^ *N*_*final*_ minus 3‰. This value should be equal to the value of the supplied plant material if the gammarids feed exclusively on plants. Uncertainties in the trophic shift < 1‰ are irrelevant because the labeling of hay that exceeded this uncertainty > 100‐fold.

In the case of cannibalism, δ^15^ *N*_*feed*_ results from the mixing of plant (i.e., leaves and hay) and animal (i.e., gammarid) food resources. The relative contribution of plant material *f*_*plant*_ was estimated by a simple mixing model based on mass balance considerations:

(4) $$\delta^{15}N_{\text{feed}}=\delta^{15}N_{\text{final}}—3‰=f_{\text{plant}}\times \delta^{15}N_{\text{plant}}+\left(1—f_{\text{plant}}\right)\times \delta^{15}N_{\text{gammarids}}$$

where *f*_*plant*_ is the fraction of plant material contributing to gammarid N and (*1—f*_*plant*_) is the fraction of cannibalized animal material contributing to N uptake. Rearranging this equation yields *f*_*plant*_:

(5) $$f_{\text{plant}}=\left(\delta^{15}N_{\text{final}}—3‰—\delta^{15}N_{\text{gammarid}}\right)/\left(\delta^{15}N_{\text{plant}}–\delta^{15}N_{\text{gammarid}}\right)$$

Inert body tissues such as the exoskeleton preserve the initial isotopic composition even after the complete turnover of metabolically active tissue. Again, a mixing model applies for the complete body:

(6) $$\delta^{15}N_{\text{body}}=f_{\text{metabolic}}\times \delta^{15}N_{\text{metabolic}}+\left(1—f_{\text{metabolic}}\right)\times \delta^{15}N_{\text{exoskeleton}}$$

After rearranging, *f*_*metabolic*_ quantifies the fraction of N in body tissue that originates from metabolic turnover rather than structural exoskeletal material and is calculated as:

(7) $$f_{\text{metabolic}}=\left(\delta^{15}N_{\text{body}}—\delta^{15}N_{\text{exoskeleton}}\right)/\left(\delta^{15}N_{\text{metabolic}}—\delta^{15}N_{\text{exoskelton}}\right)$$

Given that δ^15^ *N*_*body*_ is equal to δ^15^ *N*_*final*_ and δ^15^ *N*_*metabolic*_ is equal to δ^15^ *N*_*plant*_ + 3‰ if only plant material is consumed, and that δ^15^ *N*_*exoskeleton*_ is equal to δ^15^ *N*_*gammarid*_, Equations (5) and (7) are almost identical except for the trophic shift, which appears in the numerator in Equation (7) while it appears in the denominator in Equation (5). It is thus uncertain whether the exoskeleton or cannibalism explains cases in which the complete body does not approach δ^15^ *N*_*plant*_ + 3‰. The exoskeleton of *Gammarus fasciatus* was found to contribute 20%–25% of the total weight (Amyot et al. 1996). Assuming a similar value for the analyzed individuals, *f*_*metabolic*_ between 75% and 100% can be explained by the exoskeleton, while lower *f*_*metabolic*_ indicates the combined effect of the exoskeleton and cannibalized animal material. The trophic shift becomes negligible in Equations (5) and (7) when δ^15^ *N*_*plant*_ differs markedly from δ^15^ *N*_*gammarid*_. We thus used hay fertilized with N highly enriched in ^15^N. In this case, 1—f is the sum of cannibalized animal material and incomplete turnover, mainly due to the exoskeleton. Equations (4) and (5) require δ^15^ *N*_*gammarid*_ as one end‐member. This value, however, changed during the experiment due to the feed intake of [or by] cannibalized gammarids before their consumption [or death]. The true values cannot be determined. We used the mean of the initial and the final δ^15^ *N*_*gammarid*_ as end‐member but also ran the calculation with the initial and the final values to determine the range of extremes, within which the true value must be.

#### 2.5.3 Data Reporting and Model Performance

Results are reported as means ±95% confidence intervals (CI). CIs were used because they do not require homoscedasticity, which is often violated when strongly labeled material is compared with natural abundances, and they allow direct comparison among values in this study, with the literature, and with theoretical expectations. The fitting of the N turnover model (Equation 7) minimized the root mean squared error between measured and predicted δ^15^N. Overall model performance was quantified using the Nash–Sutcliffe efficiency (Nash and Sutcliffe 1970), which in this context corresponds to the coefficient of determination (*r* ^2^).

## 3 Results

### 3.1 Uptake of Biomass and N

To test the preferential uptake of hay over alder (H2) by *G. fossarum*, the loss of plant material over the experiment was compared between treatments. From fine‐mesh litter bags not accessible by the gammarids, 40% ± 2% of weight was lost, without significant differences between hay and alder; 40% ± 1% was lost from alder in coarse‐mesh litter bags; and 46% ± 2% was lost from coarse‐mesh hay litter bags. After 14 days of exposure, the mean C:N ratio of hay increased significantly from 24:1 (± 1) to 32:1 (± 2) (Table 1). Due to limited sample replication for alder, changes in C:N could not be robustly assessed, though observed values decreased from 22:1 (±9) to 16:1 (±1). The N content of the alder leaves remained unchanged at 2.5% ± 0.3% and that of hay decreased from 1.8% ± 0.1% to 1.4% ± 0.1%.

**TABLE 1:** Mean ± confidence interval (CI) for parameters measured at the start and the end of the experiment.

Variable	Treatment	Mean ± 95% CI on Day 0	Mean ± 95% CI on Day 14	Mean loss ± 95% CI
Plant material dry weight (mg)	Coarse litter bags			
	Alder	4020 ± 43	2412 ± 65	40% ± 1%
	Hay	4016 ± 39	2161 ± 94	46% ± 2%
	Fine litter bags			
	Alder	4003 ± 12	2410 ± 148	40% ± 4%
	Hay	4017 ± 31	2407 ± 155	40% ± 4%
Mean gammarid number	Alder	34.0 ± 0.0	25.6 ± 3.2	25% ± 10%
	Hay	34.0 ± 0.0	24.1 ± 5.1	29% ± 15%
Mean gammarid weight (mg)	Alder	2.3 ± 0.2	2.3 ± 0.5	
	Hay	2.2 ± 0.1	2.1 ± 0.4	
C:N ratio in plant material	Alder	21.6 ± 8.8	16.9 ± 0.8	
	Hay	23.6 ± 1.1	32.1 ± 1.8	
N content in gammarids (%)	Alder	9.1 ± 2.5	8.2 ± 0.5	
	Hay	9.1 ± 2.5	8.2 ± 0.1	
δ ^15^N in plant material (‰)	Alder	−1.30 ± 0.28		
	Hay	813.95^a^		
δ ^15^N in gammarids (‰)	Alder	7.30 ± 0.69	7.91 ± 0.67	
	Hay	7.30 ± 0.69	61.24 ± 18.27	

^a^ The value was taken from Floßmann et al. (2025), who provided the hay material but reported no CIs. The same method and equipment in the same lab were used in both studies.

During the 14‐day experiment, 27% ± 8% of *G. fossarum* individuals disappeared, regardless of the food resource. The loss of individuals was equivalent to the loss in total weight and N mass, given the unchanged individual weight (mean = 2.3 ± 0.1 mg) and unchanged N content (8.3% ± 0.3%) (Table 1; the CI reported here is smaller than in Table 1 because both groups were combined here). No growth occurred during the experiment: on Day 0, mean body dry mass for gammarids was 2.3 ± 0.2 mg and 2.2 ± 0.1 mg for the alder and hay treatments, respectively, and on Day 14, it was 2.3 ± 0.5 mg and 2.3 ± 0.4 mg, respectively. CIs were larger on Day 14 due to the smaller sample size compared to Day 0.

### 3.2 Body Turnover and N Accumulation in *G. fossarum*

To quantify assimilation of N from plant material and test H1, we compared δ^15^N values of each food resource and gammarids on Day 0 and Day 14. Initially, there was ~100× more N present as plant material (197 ± 1 mg compartment^−1^ in the alder flume and 145 ± 1 mg compartment^−1^ in the hay flume) than in the gammarids (2.0 ± 1.1 mg compartment^−1^ in the alder flume and 1.8 ± 0.8 mg compartment^−1^ in the hay flume), providing ample N for the gammarids. Approximately 40% from the alder to 60% from the hay was lost within the 14 days (Table 2), with the greater N loss in the hay flume reflecting the decrease of N content in hay and the slightly larger loss from the coarse‐mesh litter bags. The N loss from the gammarids was 25% in 14 days. The decrease followed an almost linear trend over time (linear regression: *R* ^2^ = 0.956, *p* < 0.001).

**TABLE 2:** Initial N masses (mg compartment^−1^) in plant material and gammarids and their changes over time (means together with their 95% intervals of confidence).

Day	Hay flume		Alder flume	
	Hay	Gammarids	Alder	Gammarids
0	145.2	6.5 ± 0.4	197.4	6.2 ± 0.6
7		5.3 ± 0.7		5.5 ± 1.0
14	56.9	3.5 ± 1.0	125.0	3.4 ± 0.9

The mean δ^15^N baseline values for starved gammarids on Day 0 were 7.30‰ ± 0.69‰. The δ^15^N value of the labeled hay was approximately 100‐fold higher (821‰). After 14 days, the δ^15^N in the gammarids in the hay flume approached a plateau, indicating that the turnover of the metabolic tissue was almost complete. The final δ^15^N of gammarids in the hay flume was 61.24‰ ± 18.27‰. Even with strong labeling, only a minor fraction of hay‐derived N (~3%) was incorporated into gammarid biomass. Considering extreme scenarios for cannibalized individuals, this ranged between 0% and 6%. Only 0.1% of the initial N in hay was assimilated by the gammarids despite a loss of 60% of N from the litter bags.

Alder leaves were isotopically closer to the initial gammarid N, with δ^15^N averaging −1.3‰ ± 0.28‰. After 14 days, gammarid δ^15^N approached a plateau, suggesting near‐complete turnover of metabolically active N. Mass balance calculations indicate that alder‐derived N contributed ~31% of total body N, highlighting a low assimilation efficiency as for hay. Due to the small δ^15^N difference between alder and gammarids, estimates of plant‐derived N are associated with higher uncertainty than for strongly labeled hay. The proportion of plant N from alder leaves was greater than from hay. The small isotopic difference between the plant material and the initial gammarids, combined with the strong effect of the trophic shift (regardless of whether Equations 5 or 7 is used), results in high uncertainty, compared to the strong difference between gammarids and labeled hay. Nevertheless, the gammarids assimilated only 0.3% of the N supplied with alder leaves.

When fitting Equation (3) to quantify turnover, the reaction progress constant was 0.10, which corresponds to a half‐life of 6.7 days. Consequently, 14 days (equivalent to 2.1 half‐lives) were sufficient to replace 76% of the metabolically active N. The final δ^15^N, as predicted by the equation, closely matched the measured values after 14 days (for the alder group, 8.13‰ vs. 7.91‰; for the hay group, 77.89‰ vs. 61.24‰). The Nash–Sutcliffe efficiency was 0.828 (*n* = 28), indicating that 83% of variation in δ^15^N was explained by the turnover model.

## 4 Discussion

**FIGURE 3:**
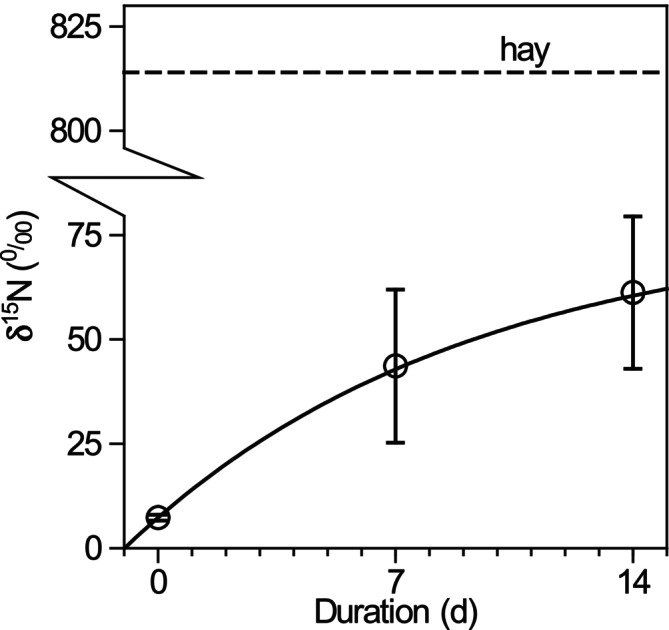
Time course of δ^15^N of *Gammarus fossarum* when fed with hay; error bars denote the 95% confidence interval (CI); the solid line is the fitted turnover equation (see text); the dashed horizontal line shows the provided hay.

**FIGURE 4:**
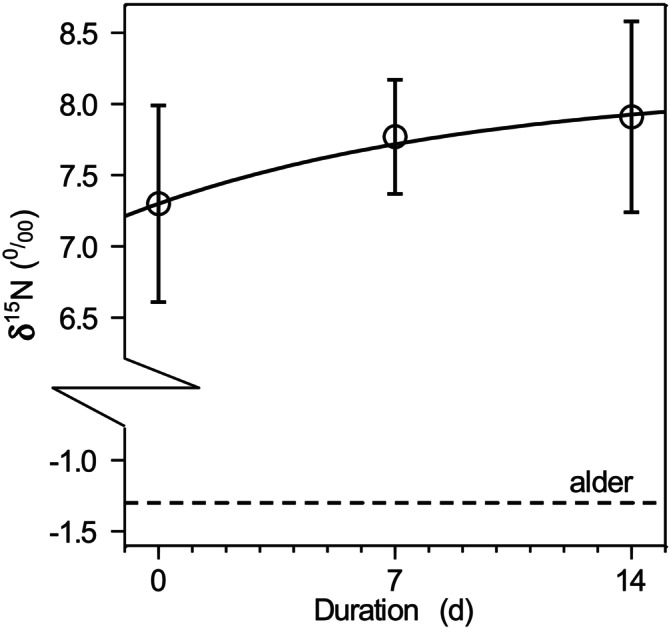
Time course of δ^15^N of *Gammarus fossarum* when fed with alder leaves; error bars denote the 95% confidence interval (CI); the solid line is the fitted turnover equation (see text); the dashed horizontal line shows the provided alder leaves.

We provide the first mechanistic quantification of N uptake from cannibalism in the common gammarid *G. fossarum* using an isotopic approach. Our results demonstrate that cannibalized animal material is a major N source for this species, which is typically classified as a shredder‐detritivore. Contrary to H1 and despite the observed degradation of plant material, assimilation of plant‐derived N by gammarids was marginal. δ^15^N values in the hay flume approached only 61.2‰ ± 18.3‰ after 14 days, far below the 824‰ expected if the complete body N were derived from hay (Figure 3). A similar pattern was observed for alder‐fed gammarids, where δ^15^N increased only slightly from the initial 7.3‰ ± 0.7‰, indicating low plant N assimilation (Figure 4). These findings reject H1.

Hypothesis H2 is also rejected: gammarids showed no preference for hay or alder leaves. Coarse‐mesh litter bags lost 46% ± 2% of hay and 40% ± 1% of alder, and mass balance calculations indicate that only 0.1% of hay‐derived N and 0.3% of alder‐derived N were incorporated into gammarid biomass (Table 1). This supports the conclusion that cannibalism dominated N uptake under our experimental conditions.

Hypothesis H3, concerning turnover differences between plant types, could not be robustly evaluated due to the low isotopic spacing between alder and gammarids and the overwhelming contribution of animal‐derived N. Nevertheless, mass balance suggests that alder‐derived N contributed approximately 31% of body N, compared to only ~3% from hay, implying slightly higher utilization of alder than hay despite strong cannibalistic feeding (Figure 4).

The high initial δ^15^N of *G. fossarum* (7.3‰) further suggests that, under natural conditions, diet includes both plant and animal material. Terrestrial plant material from local grasslands exhibits much lower δ^15^N (2.3‰ ± 2.1‰; Schwertl et al. 2005), even after accounting for trophic enrichment of 3‰. Thus, a substantial portion of N in gammarids in natural habitats must originate from animal‐derived sources, highlighting their dual role as detritivores and predators. Comparable patterns have been observed in other gammarids, for example, *Dikerogammarus villosus* with δ^15^N = 11.5‰ (Brandner et al. 2013), and predation on macroinvertebrates by *Gammarus* species is well documented (Kelly et al. 2002b; MacNeil et al. 1997; Syrovátka et al. 2020). Laboratory studies also report cannibalism, especially targeting recently molted or smaller individuals (Alther et al. 2023; Dick and Platvoet 1996; McGrath et al. 2007).

Our study emphasizes that cannibalism can substantially alter trophic position assessments in gammarids. Standard use of gammarids as model shredders or as primary‐consumer baselines may underestimate their reliance on animal‐derived N. The δ^15^N data indicate that up to 70% of assimilated N could originate from cannibalized conspecifics, while only a maximum of 33% could be attributed to alder leaves. Controlled experiments or stable isotope studies that ignore cannibalism may therefore misrepresent the contribution of plant material to gammarid diets (Kühmayer et al. 2020; Syrovátka et al. 2020).

We could not distinguish whether cannibalized individuals were actively preyed upon or consumed opportunistically, since the outcome for N assimilation is equivalent. The short exposure time and disappearance rate support the likelihood of active predation, consistent with previous observations (Kelly et al. 2002b; McGrath et al. 2007). Cannibalism was unlikely to be induced by density, size variation, or food limitation: gammarid densities were comparable to natural conditions (Alther et al. 2024), individuals were size‐matched and starved for only 24 h, and plant N availability exceeded body N by ~100‐fold. Minor differences in biomass loss between coarse‐ and fine‐mesh litter bags likely reflect handling effects rather than feeding preferences.

Turnover rates of N were slower than expected from the high δ^15^N enrichment of hay. This is partly attributable to the exoskeleton acting as an inert N pool (20%–25% of total body mass; Amyot et al. 1996), but the limited assimilation of plant N despite high labeling indicates reliance on animal‐derived N. Equation (3) predicts that 76% of metabolically active N was replaced after 14 days (half‐life = 6.7 days), yet δ^15^N values confirm minimal uptake from hay (Figure 3).

Finally, our findings have ecological implications. Gammarids' realized trophic position likely depends on resource availability and prey abundance. Under conditions of low‐quality detritus or abundant juvenile prey, *G. fossarum* may increase predation, occupying a higher trophic position. In contrast, we cannot exclude that under high‐quality detrital input, detritivory may dominate (Khaliq et al. 2025). Isotopic tracer studies, field experiments, and mesocosm approaches incorporating labeled plant and animal sources could further refine our understanding of gammarid feeding behavior and nutrient fluxes in stream food webs.

## 5 Conclusion

Our findings demonstrate that *G. fossarum* relies heavily on animal‐derived N, including cannibalized conspecifics, while assimilation of plant‐derived N is minimal. This emphasizes the species' functional role as an omnivorous consumer in stream food webs, influencing both detritus processing and nutrient transfer. Studies using gammarids as model shredders or primary‐consumer baselines should account for their predatory behavior to avoid misrepresenting ecosystem functions. Future experiments with isotopically labeled animal and plant sources in mesocosms or field settings will help refine the understanding of their functional contribution to nutrient dynamics.

## Author Contributions

**Katharina Amann:** data curation (equal), formal analysis (equal), writing – original draft (lead), writing – review and editing (equal). **Rebecca Hoess:** conceptualization (equal), data curation (equal), methodology (equal), writing – review and editing (equal). **Karl Auerswald:** formal analysis (equal), supervision (equal), visualization (lead), writing – review and editing (equal). **Juergen Geist:** conceptualization (equal), funding acquisition (lead), methodology (equal), resources (lead), supervision (lead), validation (equal), writing – review and editing (equal).

## Funding

This work was supported by the project “Bayerische Landschaften im Klimawandel II [TKP01KPB‐78573]” and by the Bayerisches Staatsministerium für Umwelt und Verbraucherschutz, München, Germany.

## Conflicts of Interest

The authors declare no conflicts of interest.

## Supporting information




Data S1.

## Data Availability

Data are provided in the Supporting Information S1.
