# Supplementary material for: Nitrogen Assimilation in the Freshwater Amphipod Gammarus fossarum is Dominated by Animal‐Derived Resources
Source: Ecol Evol. 2026 Apr 21;16(4):e73556. doi: 10.1002/ece3.73556 (PMC13099149; doi:10.1002/ece3.73556)
Supplement: Supplementary file 1 — Data S1. [file ECE3-16-e73556-s001.pdf]

Supplement to:

Nitrogen assimilation in the freshwater amphipod *Gammarus fossarum* is dominated by animal-derived resources

Katharina Amann, Rebecca Hoess, Karl Auerswald, Juergen Geist*

Aquatic Systems Biology Unit, TUM School of Life Sciences, Technical University of Munich, Mühlenweg 22, 85354 Freising, Germany

* geist@tum.de

Overview:

Table 1: Allometric data of body length and body weight for *Gammarus fossarum* Page 2

Table 2: Nitrogen masses in plant material and *Gammarus fossarum* Page 12

Table 3: Group mean $\delta^{15}N$ of *Gammarus fossarum* Page 13

Table 4: Mortality (losses) of *Gammarus fossarum* during the experiment Page 14

Table 5: Specimen $\delta^{15}N$ and nitrogen content of *Gammarus fossarum* Page 15

Table 6: Weight and length at day 0, 7 and 14 Page 16

Table 1: Allometric relation between body length and dry weight of *Gammarus fossarum*. Data were obtained at day 0, 7 and 14. Note: dry weight could only be determined at day 7 and 14 for the specimen used in the experiment while wet weight was determined at day 0.

Day	Length (mm)	Wet weight (mg)	Dry weight (mg)	Day	Length (mm)	Wet weight (mg)	Dry weight (mg)
0	7.938	12.1	2.1	0	8.813	13.4	
0	8.864	13.5	2.9	0	7.895	9.5	
0	8.357	16.4	2.3	0	8.179	8.8	
0	5.903	5.2	0.6	0	5.438	4.3	
0	8.008	14.0	2.3	0	8.072	10.0	
0	9.298	19.9	4.7	0	10.148	19.7	
0	5.74	7.8	1.3	0	8.71	13.4	
0	5.655	4.9	1.3	0	8.822	11.2	
0	7.011	7.4	1.5	0	9.299	25.3	
0	8.126	16.2	3.3	0	9.248	12.8	
0	6.646	9.6	1.6	0	10.316	16.6	
0	6.653	6.9	1.7	0	10.933	29.9	
0	7.499	17.7	2.7	0	7.965	10.7	
0	6.074	7.5	1.1	0	8.894	13.2	
0	7.546	12.8	3.2	0	9.058	16.4	
0	5.065	2.8	0.8	0	6.784	9.6	
0	8.61	12.7	2.4	0	7.689	8.3	
0	7.415	11.6	2.0	0	8.2	9.1	
0	5.346	5.5	1.1	0	7.336	8.7	
0	9.868	17.7		0	7.762	13.5	
0	12.271	30.5		0	8.324	10.2	
0	6.194	6.6		0	8.091	11.7	
0	8.291	12.9		0	7.3	12.6	
0	10.05	18.4		0	6.871	10.8	
0	7.61	10.9		0	7.502	3.2	
0	7.105	10.7		0	8.317	8.6	
0	8.653	12.6		0	6.31	4.3	
0	11.129	22.5		0	9.939	23.9	
0	6.518	6.8		0	10.552	16.6	
0	10.214	16.5		0	8.66	12.1	
0	10.841	21.5		0	8.31	10.4	
0	7.956	8.8		0	7.033	7.6	
0	9.954	18.5		0	9.181	14.6	
0	6.076	4.6		0	7.582	10.4	
0	8.344	10.4		0	6.162	5.2	
0	8.247	11.8		0	7.628	6.4	
0	8.679	10.7		0	8.06	7.8	
0	6.7	6.5		0	8.139	9.8	
0	6.741	5.8		0	7.285	5.6	
0	6.901	5.3		0	8.57	13.0	

0	6.578	8.2
0	10.111	18.5
0	10.152	14.9
0	10.265	9.2
0	8.917	12.6
0	6.429	7.6
0	6.607	6.8
0	8.906	8.7
0	6.418	9.2
0	7.851	4.2
0	6.31	6.1
0	7.993	9.4
0	8.897	11.4
0	8.414	10.7
0	11.081	21.5
0	10.088	18.1
0	7.303	7.9
0	8.721	9.6
0	7.11	7.6
0	9.597	17.0
0	6.683	4.6
0	8.762	9.1
0	7.07	8.6
0	8.416	10.6
0	8.539	10.2
0	7.636	6.4
0	9.653	14.3
0	9.18	15.3
0	7.649	6.0
0	7.652	8.1
0	10.052	13.1
0	7.651	7.3
0	9.415	10.5
0	6.976	4.2
0	6.842	7.6
0	6.431	6.5
0	12.562	28.0
0	12.001	28.5
0	9.803	15.2
0	8.044	8.7
0	7.258	7.1
0	7.198	6.9
0	6.121	8.5
0	7.748	7.2
0	5.928	4.6
0	10.847	20.8
0	7.28	8.1

0	11.047	20.8
0	9.16	22.2
0	10.424	17.8
0	11.105	18.4
0	8.264	10.5
0	9.207	11.2
0	10.188	20.5
0	8.026	9.8
0	9.274	14.9
0	8.897	11.8
0	9.641	18.4
0	8.003	16.9
0	9.385	12.8
0	8.758	10.2
0	7.541	8.3
0	7.542	16.7
0	8.104	12.3
0	6.424	6.6
0	9.414	17.5
0	8.314	5.6
0	7.28	7.6
0	8.45	11.4
0	10.774	24.4
0	7.693	6.7
0	6.68	7.3
0	9.776	18.2
0	7.922	7.8
0	8.531	15.4
0	7.85	7.5
0	7.239	5.8
0	6.625	7.0
0	6.354	3.8
0	7.938	12.1
0	8.864	13.5
0	8.357	16.4
0	5.903	5.2
0	8.008	14.0
0	9.298	19.9
0	5.74	7.8
0	5.655	4.9
0	7.011	7.4
0	8.126	16.2
0	6.646	9.6
0	6.653	6.9
0	7.499	17.7
0	6.074	7.5
0	7.546	12.8

0	10.127	20.9
0	9.496	13.9
0	8.449	10.3
0	9.284	14.1
0	11.329	28.8
0	11.519	19.9
0	8.128	11.0
0	7.283	6.4
0	6.769	4.9
0	10.626	20.0
0	9.77	14.0
0	8.376	9.4
0	7.497	7.8
0	10.537	14.6
0	6.418	6.5
0	7.366	5.7
0	7.275	5.3
0	7.799	9.1
0	9.17	11.7
0	6.645	7.4
0	9.663	17.1
0	6.935	3.7
0	8.184	12.2
0	9.75	17.7
0	9.67	15.6
0	7.783	6.8
0	7.447	16.9
0	8.591	16.2
0	9.432	6.6
0	8.506	15.5
0	8.144	5.6
0	8.17	6.7
0	8.196	10.6
0	9.727	16.1
0	7.482	7.1
0	8.311	10.1
0	8.047	9.3
0	6.996	7.4
0	8.264	16.9
0	8.128	11.4
0	9.738	12.8
0	6.523	7.7
0	8.817	14.4
0	7.759	6.8
0	11.767	29.4
0	10.884	21.0
0	9.676	15.8

0	5.065	2.8	
0	8.61	12.7	
0	7.415	11.6	
0	5.346	5.5	
7	9.251		3.2
7	8.63		2.5
7	9.209		2.7
7	7.205		0.9
7	12.059		5.9
7	7.541		2.0
7	9.275		3.2
7	9.899		3.8
7	8.802		2.5
7	7.301		1.7
7	8.178		2.1
7	9.641		4.2
7	7.659		2.2
7	9.482		3.3
7	8.815		2.2
7	11.874		5.7
7	6.549		0.4
7	8.007		1.8
7	6.245		1.6
7	10.3		5.7
7	8.931		2.9
7	8.495		2.2
7	8.69		3.7
7	7.073		1.9
7	8.268		1.9
7	10.386		4.2
7	7.812		2.1
7	6.487		1.4
7	8.148		2.3
7	9.586		3.0
7	7.371		1.6
7	7.06		0.9
7	8.399		2.1
7	10.13		3.5
7	8.864		2.4
7	9.74		3.2
7	10.022		3.9
7	7.94		1.6
7	8.798		2.2
7	9.72		3.1
7	8.684		2.3
7	8.149		2.3
7	7.615		1.4

0	7.105	17.7	7	7.293	1.6
0	8.689	10.5	7	9.982	3.5
0	7.051	6.8	7	7.246	1.3
0	9.294	15.5	7	7.734	1.8
0	8.115	12.7	7	7.602	1.4
0	9.96	27.1	7	8.143	2.5
0	8.928	12.1	7	7.81	2.3
0	7.608	5.6	7	8.332	2.2
0	7.235	6.7	7	7.947	2.2
0	9.724	18.6	7	10.679	4.7
0	7.681	8.0	7	7.004	1.3
0	6.913	7.3	7	9.124	2.0
0	9.142	11.0	7	8.571	1.2
0	6.138	5.8	7	10.446	4.1
0	7.591	3.8	7	10.707	4.4
0	7.332	9.4	7	10.405	3.6
0	6.657	6.0	7	9.038	2.1
0	6.77	8.0	7	6.522	1.3
0	6.78	6.9	7	6.11	1.1
0	6.549	9.2	7	7.375	1.5
0	6.61	1.3	7	7.379	1.4
0	8.833	12.5	7	8.523	1.9
0	7.967	7.8	7	8.537	2.7
0	8.831	13.8	7	8.422	2.4
0	11.196	21.6	7	8.273	1.9
0	9.03	13.2	7	10.446	3.5
0	8.623	12.1	7	10.707	3.8
0	5.914	4.1	7	10.405	
0	8.758	10.2	7	9.038	2.7
0	7.122	4.2	7	6.522	0.4
0	9.446	13.7	7	6.11	0.2
0	10.332	18.9	7	7.375	1.6
0	8.391	12.5	7	7.379	1.3
0	6.459	4.2	7	8.523	2.1
0	8.678	14.1	7	8.537	2.0
0	7.014	3.8	7	8.422	1.7
0	10.108	15.6	7	8.273	1.3
0	7.954	9.9	7	9.668	4.3
0	9.634	13.0	7	7.445	2.6
0	6.413	7.7	7	7.063	2.5
0	7.079	5.7	7	8.566	3.0
0	7.304	8.1	7	9.479	4.1
0	8.529	17.7	7	10.626	5.4
0	6.71	15.6	7	7.466	3.3
0	8.397	9.7	7	6.983	2.4
0	10.466	10.1	7	7.555	1.8
0	8.09	11.1	7	7.898	3.8

0	9.637	8.6	7	6.99	1.9
0	8.263	6.2	7	6.605	1.4
0	9.659	3.4	7	7.646	1.8
0	7.895	14.4	7	8.632	2.4
0	7.16	6.8	7	8.585	2.3
0	8.931	16.7	7	8.277	2.4
0	7.739	10.8	7	9.941	3.3
0	7.917	11.0	7	7.179	1.3
0	8.533	11.1	7	8.037	2.1
0	9.25	16.6	7	9.609	2.8
0	6.503	4.1	7	6.8	1.5
0	11.713	26.2	7	7.574	1.5
0	6.147	4.8	7	8.47	1.8
0	10.937	16.4	7	9.215	3.8
0	8.085	9.9	7	7.486	2.0
0	10.57	17.3	7	8.137	2.1
0	7.191	6.6	7	10.028	3.6
0	5.886	3.9	7	7.299	1.5
0	8.433	13.6	7	7.6	2.0
0	8.522	12.2	7	10.414	3.8
0	7.365	6.8	7	9.029	2.8
0	8.204	8.4	7	9.052	2.9
0	5.981	5.2	7	10.5	4.0
0	6.06	5.2	7	10.327	3.9
0	6.399	5.5	7	10.297	4.0
0	8.59	16.2	7	8.987	3.4
0	6.157	3.5	7	7.809	1.9
0	6.85	7.0	7	8.936	3.7
0	7.908	8.4	7	7.017	1.8
0	7.972	8.0	7	9.856	4.0
0	9.098	12.4	7	8.233	2.3
0	7.66	8.0	7	10.267	4.7
0	9.282	18.2	7	8.561	2.5
0	8.527	9.8	7	8.914	3.2
0	6.711	4.0	7	8.705	2.8
0	11.865	23.1	7	8.58	1.9
0	7.971	11.6	7	7.392	1.3
0	6.543	7.2	7	8.195	2.6
0	6.375	12.5	7	8.94	2.4
0	8.214	8.1	7	7.104	1.6
0	6.58	14.6	7	7.489	1.4
0	8.477	17.4	7	10.272	4.2
0	9.951	5.6	7	9.716	3.7
0	5.906	9.4	7	9.626	2.8
0	8.059	3.4	7	7.71	1.9
0	5.19	4.9	7	9.51	3.2
0	6.481	7.7	7	8.929	2.1

0	7.627	6.6	7	9.11	3.1
0	7.703	6.1	7	6.919	1.0
0	6.911	6.8	14	9.35	2.9
0	7.268	12.6	14	11.458	2.4
0	8.256	6.8	14	7.813	1.9
0	6.552	4.8	14	6.847	1.5
0	7.089	7.7	14	6.974	1.1
0	7.684	6.0	14	8.987	2.0
0	7.248	7.7	14	8.165	1.9
0	7.221	13.6	14	7.795	2.0
0	8.987	13.6	14	10.182	4.0
0	7.934	7.9	14	8.667	2.1
0	7.666	6.8	14	10.411	3.1
0	7.358	6.8	14	11.574	3.9
0	7.534	8.7	14	7.883	1.6
0	7.326	4.3	14	9.462	3.2
0	5.835	16.4	14	7.797	1.4
0	9.987	16.3	14	11.592	6.0
0	10.493	6.2	14	7.344	1.8
0	6.363	16.9	14	5.836	0.7
0	11.019	21.5	14	6.663	0.9
0	8.674	13.7	14	7.36	1.4
0	7.697	7.0	14	7.088	0.8
0	7.611	9.4	14	12.313	6.7
0	8.921	10.8	14	10.094	3.8
0	7.122	6.2	14	10.62	3.5
0	7.474	8.0	14	6.651	1.4
0	7.62	7.5	14	10.555	3.4
0	7.761	8.1	14	10.29	4.5
0	8.288	18.6	14	10.95	4.3
0	8.408	19.5	14	7.958	1.9
0	10.732	17.3	14	8.871	2.9
0	8.038	16.6	14	8.404	2.4
0	8.489	10.1	14	9.464	3.2
0	8.269	10.2	14	8.111	2.5
0	7.399	11.6	14	6.935	1.1
0	8.137	11.0	14	10.241	3.4
0	7.724	9.4	14	7.327	1.4
0	9.752	7.9	14	8.018	1.4
0	7.308	13.0	14	7.702	1.9
0	7.89	12.0	14	7.618	1.3
0	8.328	8.8	14	8.456	2.1
0	8.586	9.3	14	7.333	1.1
0	8.038	10.1	14	8.15	1.7
0	7.985	7.7	14	7.285	1.1
0	8.485	9.6	14	11.209	4.5
0	8.54	11.4	14	6.805	1.5

0	8.672	10.7	14	10.099	4.5
0	8.572	26.4	14	8.762	2.7
0	8.41	11.3	14	6.984	1.8
0	7.443	11.4	14	7.741	1.6
0	6.277	8.2	14	8.031	1.8
0	9.521	15.4	14	6.45	1.2
0	10.453	21.9	14	6.646	1.7
0	9.027	12.1	14	8.726	2.7
0	7.061	7.9	14	10.205	3.1
0	6.492	5.7	14	9.306	2.4
0	6.79	6.2	14	7.025	1.7
0	8.002	9.1	14	7.6	1.7
0	6.962	7.5	14	8.005	1.0
0	8.048	19.1	14	10.922	2.1
0	10.409	19.0	14	7.036	1.6
0	9.854	14.5	14	6.097	0.4
0	7.721	8.8	14	7.512	1.6
0	7.833	6.8	14	7.308	1.6
0	8.42	11.0	14	7.161	2.0
0	7.569	6.9	14	6.308	1.4
0	8.533	11.0	14	8.398	2.0
0	7.466	7.9	14	8.722	2.8
0	8.856	14.5	14	7.586	1.4
0	9.542	19.6	14	8.932	2.8
0	8.833	14.6	14	9.435	2.6
0	8.409	11.1	14	8.317	2.2
0	9.861	12.6	14	8.909	2.0
0	9.307	18.7	14	11.363	4.9
0	6.538	7.9	14	8.368	2.4
0	6.877	7.5	14	7.759	1.9
0	8.407	6.0	14	8.999	3.0
0	7.264	5.6	14	9.388	3.8
0	6.562	6.5	14	7.925	1.6
0	7.141	5.8	14	7.586	1.8
0	7.327	9.6	14	7.437	2.1
0	9.987	13.4	14	7.384	1.6
0	10.551	17.7	14	9.6	2.7
0	9.106	15.4	14	8.643	2.5
0	8.555	13.8	14	6.736	1.4
0	8.107	10.0	14	8.471	1.3
0	11.388	22.9	14	7.144	1.4
0	9.167	13.8	14	9.332	3.5
0	6.866	6.8	14	7.316	1.7
0	6.875	4.8	14	10.136	3.5
0	7.308	3.5	14	8.267	2.5
0	9.186	15.9	14	6.98	1.2
0	7.992	7.4	14	9.227	4.9

0	7.794	13.8	14	9.282	2.7
0	9.089	11.2	14	8.149	2.6
0	11.4	26.9	14	9.115	3.3
0	7.903	9.8	14	8.091	2.3
0	9.502	17.6	14	6.423	1.4
0	9.088	16.6	14	8.836	2.4
0	8.454	15.6	14	6.993	1.5
0	9.049	20.9	14	9.841	3.5
0	7.137	7.7	14	7.33	1.9
0	6.863	3.0	14	7.701	2.5
0	10.639	35.5	14	7.24	1.2
0	9.516	13.3	14	8.755	2.4
0	9.49	10.6	14	8.37	2.3
0	7.949	6.7	14	9.514	3.0
0	8.324	18.5	14	7.363	1.0
0	8.143	10.3	14	9.594	2.4
0	9.084	14.6	14	6.536	1.3
0	7.683	26.1	14	7.281	1.9
0	6.942	4.1	14	10.006	3.6
0	6.832	7.2	14	10.177	4.1
0	8.286	12.2	14	9.725	1.6
0	6.409	5.9	14	7.211	1.7
0	6.306	7.6	14	8.535	2.4
0	12.275	25.0	14	6.645	1.4
0	6.162	6.3			
0	8.159	9.8			
0	8.445	11.3			

Table 2: Initial N masses (mg compartment^{-1}) in plant material and gammarids and their changes over time (means together with their 95% intervals of confidence)

Day	Hay flumes		Alder flumes	
	Hay	Gammarids	Alder	Gammarids
0	145.2	6.5 ± 0.4	197.4	6.2 ± 0.6
7		5.3 ± 0.7		5.5 ± 1.0
14	56.9	3.5 ± 1.0	125.0	3.4 ± 0.9

Table 3: Time course of δ^{15}N of *Gammarus fossarum* when fed with either hay or alder leaves; error bars denote the 95% confidence interval

	Gammarids fed with hay		Gammarids fed with alder	
Day	Mean	95% confidence interval	Mean	95% confidence interval
0	7.30	0.69	7.30	0.69
7	43.61	18.35	7.77	0.40
14	61.24	18.27	7.91	0.67

Table 4: Losses of specimen (mortality) during the experiment

Compartment	Initial abundance	Removed after day 7	Present at day 14	Deaths
Alder 1	32	11	13	8
Alder 2	32	10	8	14
Alder 3	32	11	13	8
Alder 4	32	12	9	11
Alder 5	32	12	10	10
Alder 6	32	12	8	12
Hay 1	32	12	6	14
Hay 2	32	12	7	13
Hay 3	32	11	18	3
Hay 4	32	12	4	16
Hay 5	32	12	12	8
Hay 6	32	12	8	12

Table 5: δ^{15}N and dry-weight N content of *Gammarus fossarum* specimen when fed with either hay or alder leaves

Compartment	Plant type	Time of the experiment (d)	δ15N (‰)	N content (%)
Alder 1	alder	7	7.68	9.22
Alder 2	alder	7	8.52	9.26
Alder 3	alder	7	7.74	9.38
Alder 4	alder	7	7.55	7.90
Alder 5	alder	7	7.60	7.88
Alder 6	alder	7	7.52	7.96
Alder 1	alder	14	7.49	7.92
Alder 2	alder	14	9.17	8.90
Alder 3	alder	14	7.67	7.91
Alder 4	alder	14	7.79	8.70
Alder 5	alder	14	7.88	7.98
Alder 6	alder	14	7.44	7.89
Hay 1	hay	7	61.39	7.44
Hay 2	hay	7	43.91	7.71
Hay 3	hay	7	57.91	7.81
Hay 4	hay	7	21.14	7.77
Hay 5	hay	7	53.71	9.00
Hay 6	hay	7	23.59	6.67
Hay 1	hay	14	53.43	8.04
Hay 2	hay	14	74.85	8.28
Hay 3	hay	14	75.69	8.43
Hay 4	hay	14	79.31	8.30
Hay 5	hay	14	42.34	8.17
Hay 6	hay	14	41.84	8.27
Initial	reference	0	7.43	8.21
Initial	reference	0	6.83	8.26
Initial	reference	0	7.12	8.37
Initial	reference	0	7.84	11.46

Table 6: Weight and length of *Gammarus fossarum* specimen at day 0, 7 and 14

	Alder Treatment						Hay Treatment					
Compartment:	1	2	3	4	5	6	1	2	3	4	5	6
Specimen No.	Wet weight at day 0 (mg)											
1	17.70	11.40	8.10	16.10	1.30	11.00	14.60	10.80	9.10	3.50	10.00	5.60
2	30.50	10.70	20.90	7.10	12.50	11.10	17.40	6.20	7.50	15.90	19.70	13.00
3	6.60	21.50	13.90	10.10	7.80	16.60	5.60	8.00	19.10	7.40	13.40	20.80
4	12.90	18.10	10.30	9.30	13.80	4.10	9.40	7.50	19.00	13.80	11.20	22.20
5	18.40	7.90	14.10	7.40	21.60	26.20	3.40	8.10	14.50	11.20	25.30	17.80
6		9.60	28.80	16.90	13.20	4.80	4.90	18.60	8.80	26.90	12.80	18.40
7	10.90	7.60	19.90	11.40	12.10	16.40	7.70	19.50	6.80	9.80	16.60	10.50
8	10.70	17.00	11.00	12.80	4.10	9.90	6.60	17.30	11.00	17.60	29.90	11.20
9	12.60	4.60	6.40	7.70	10.20	17.30	6.10	16.60	6.90	16.60	10.70	20.50
10	22.50	9.10	4.90	14.40	4.20	6.60	6.80	10.10	11.00	15.60	13.20	9.80
11	6.80	8.60	20.00	6.80	13.70	3.90	12.60	10.20	7.90	20.90	16.40	14.90
12	16.50	10.60	14.00	29.40	18.90	13.60	6.80	11.60	14.50	7.70	9.60	11.80
13	21.50	10.20	9.40	21.00	12.50	12.20		11.00	19.60	3.00	8.30	18.40
14	8.80	6.40	7.80	15.80	4.20	6.80	4.80	9.40	14.60	35.50	9.10	16.90
15	18.50	14.30	14.60	17.70	14.10	8.40	7.70	7.90	11.10	13.30	8.70	12.80
16	4.60	15.30	6.50	10.50	3.80	5.20	6.00	13.00	12.60	10.60	13.50	10.20
17	10.40	6.00	5.70	6.80	15.60	5.20	7.70	12.00	18.70	6.70	10.20	8.30
18	11.80	8.10	5.30	15.50	9.90	5.50	13.60	8.80	7.90	18.50	11.70	16.70
19	10.70	13.10	9.10	12.70	13.00	16.20	13.60	9.30	7.50	10.30	12.60	12.30
20	6.50	7.30	17.10	27.10	7.70	3.50	7.90	10.10	6.00	14.60	10.80	6.60
21	5.80	10.50	7.40	12.10	5.70	7.00	6.80	7.70	5.60	26.10	3.20	17.50
22	5.30	4.20	17.10	5.60	8.10	8.40	6.80	9.60	6.50	4.10	8.60	5.60
23	8.20	7.60	3.70	6.70	17.70	8.00	8.70	11.40	7.60	7.20	4.30	7.60
24	18.50	6.50	12.20	18.60	15.60	12.40	4.30	10.70	9.60	12.20	23.90	11.40
25	14.90	28.00	17.70	8.00	9.70	8.00	16.40	26.40	13.40	5.90	16.60	24.40
26	7.60	28.50	15.60	7.30	10.10	18.20	16.30	11.30	17.70	7.60	12.10	6.70
27	12.60	15.20	6.80	11.00	11.10	9.80	6.20	11.40	15.40	25.00	10.40	7.30
28	7.60	8.70	16.90	5.80	8.60	4.00	16.90	8.20	13.80	6.30	7.60	18.20
29	6.80	7.10	16.20	3.80	6.20	23.10	21.50	15.40	13.30	9.80	14.60	7.80
30	8.70	6.90	6.60	9.40	3.40	11.60	13.70	21.90	10.00	11.30	10.40	15.40
31	9.20	8.50	15.50	6.00	14.40	7.20	7.00	12.10	22.90	13.40	5.20	7.50
32	4.20	7.20	5.60	8.00	6.80	12.50	9.40	7.90	13.80	9.50	6.40	5.80
33	6.10	4.60	6.70	6.90	16.70	8.10	3.10	5.70	6.80	8.80	7.80	7.00
34	9.40	20.80	10.60	9.20	10.80	5.30		6.20	4.80	4.30	9.80	3.80

Table 6: continued

	Alder Treatment						Hay Treatment					
Compartment:	1	2	3	4	5	6	1	2	3	4	5	6
	Length at day 0 (mm)											
1	9.87	8.90	7.28	9.73	6.61	7.92	8.48	8.92	8.00	7.31	8.07	7.29
2	12.27	8.41	10.13	7.48	8.83	8.53	9.95	7.12	6.96	9.19	10.15	8.57
3	6.19	11.08	9.50	8.31	7.97	9.25	5.91	7.47	8.05	7.99	8.71	11.05
4	8.29	10.09	8.45	8.05	8.83	6.50	8.06	7.62	10.41	7.79	8.82	9.16
5	10.05	7.30	9.28	7.00	11.20	11.71	5.19	7.76	9.85	9.09	9.30	10.42
6	7.95	8.72	11.33	8.26	9.03	6.15	6.48	8.29	7.72	11.40	9.25	11.11
7	7.61	7.11	11.52	8.13	8.62	10.94	7.63	8.41	7.83	7.90	10.32	8.26
8	7.11	9.60	8.13	9.74	5.91	8.09	7.70	10.73	8.42	9.50	10.93	9.21
9	8.65	6.68	7.28	6.52	8.76	10.57	6.91	8.04	7.57	9.09	7.97	10.19
10	11.13	8.76	6.77	8.82	7.12	7.19	7.27	8.49	8.53	8.45	8.89	8.03
11	6.52	7.07	10.63	7.76	9.45	5.89	8.26	8.27	7.47	9.05	9.06	9.27
12	10.21	8.42	9.77	11.77	10.33	8.43	7.59	7.40	8.86	7.14	6.78	8.90
13	10.84	8.54	8.38	10.88	8.39	8.52	6.55	8.14	9.54	6.86	7.69	9.64
14	7.96	7.64	7.50	9.68	6.46	7.37	7.09	7.72	8.83	10.64	8.20	8.00
15	9.95	9.65	10.54	7.11	8.68	8.20	7.68	9.75	8.41	9.52	7.34	9.39
16	6.08	9.18	6.42	8.69	7.01	5.98	7.25	7.31	9.86	9.49	7.76	8.76
17	8.34	7.65	7.37	7.05	10.11	6.06	7.22	7.89	9.31	7.95	8.32	7.54
18	8.25	7.65	7.28	9.29	7.95	6.40	8.99	8.33	6.54	8.32	8.09	7.54
19	8.68	10.05	7.80	8.12	9.63	8.59	7.93	8.59	6.88	8.14	7.30	8.10
20	6.70	7.65	9.17	9.96	6.41	6.16	7.67	8.04	8.41	9.08	6.87	6.42
21	6.74	9.42	6.65	8.93	7.08	6.85	7.36	7.99	7.26	7.68	7.50	9.41
22	6.90	6.98	9.66	7.61	7.30	7.91	7.53	8.49	6.56	6.94	8.32	8.31
23	6.58	6.84	6.94	7.24	8.53	7.97	7.33	8.54	7.14	6.83	6.31	7.28
24	10.11	6.43	8.18	9.72	6.71	9.10	5.84	8.67	7.33	8.29	9.94	8.45
25	10.15	12.56	9.75	7.68	8.40	7.66	9.99	8.57	9.99	6.41	10.55	10.77
26	10.27	12.00	9.67	6.91	10.47	9.28	10.49	8.41	10.55	6.31	8.66	7.69
27	8.92	9.80	7.78	9.14	8.09	8.53	6.36	7.44	9.11	12.28	8.31	6.68
28	6.43	8.04	7.45	6.14	9.64	6.71	11.02	6.28	8.56	6.16	7.03	9.78
29	6.61	7.26	8.59	7.59	8.26	11.87	8.67	9.52		8.16	9.18	7.92
30	8.91	7.20	9.43	7.33	9.66	7.97	7.70	10.45	8.11	8.45	7.58	8.53
31	6.42	6.12	8.51	6.66	7.90	6.54	7.61	9.03	11.39	8.81	6.16	7.85
32	7.85	7.75	8.14	6.77	7.16	6.38		7.06	9.17	7.90	7.63	7.24
33	6.31	5.93	8.17	6.78	8.93	8.21	6.72	6.49	6.87	8.18	8.06	6.63
34	7.99	10.85	8.20	6.55	7.74	6.58		6.79	6.88	5.44	8.14	6.35

Table 6: continued

	Alder Treatment						Hay Treatment					
Compartment:	1	2	3	4	5	6	1	2	3	4	5	6
	Dry weight at day 7 (mg)											
1	3.23	4.23	2.22	2.05	3.49	4.07	3.48	4.26	1.78	3.75	3.42	2.56
2	2.51	2.24	3.67	3.54	1.28	4.37	3.78	2.60	2.37	1.96	1.87	2.41
3	2.66	3.28	1.85	2.44	1.78	3.59		2.53	2.33	2.09	3.68	1.56
4	0.94	2.18	1.87	3.18	1.43	2.13	2.73	2.99	2.41	3.64	1.82	1.44
5	5.88	5.71	4.19	3.92	2.51	1.34	0.42	4.11	3.34	1.49	4.02	4.19
6	2.03	0.44	2.11	1.60	2.26	1.10	0.23	5.35	1.27	1.95	2.25	3.69
7	3.23	1.82	1.36	2.20	2.18	1.53	1.59	3.30	2.09	3.84	4.69	2.82
8	3.78	1.63	2.34	3.13	2.16	1.41	1.33	2.44	2.76	2.82	2.53	1.90
9	2.50	5.70	3.04	2.29	4.69	1.88	2.10	1.78	1.52	2.93	3.23	3.24
10	1.71	2.89	1.56	2.25	1.33	2.70	1.97	3.81	1.52	4.00	2.78	2.11
11	2.07		0.89	1.42	1.95	2.41	1.69	1.92	1.80	3.91	1.94	3.10
12				1.62	1.16	1.89	1.27	1.37		3.96	1.34	0.99

Table 6: continued

	Alder Treatment						Hay Treatment					
Compartment:	1	2	3	4	5	6	1	2	3	4	5	6
	Length at day 7 (mm)											
1	9.25	9.64	8.50	8.40	9.98	10.45	10.45	9.67	7.65	9.22	8.99	8.20
2	8.63	7.66	8.69	10.13	7.25	10.71	10.71	7.45	8.63	7.49	7.81	8.94
3	9.21	9.48	7.07	8.86	7.73	10.41	10.41	7.06	8.59	8.14	8.94	7.10
4	7.21	8.82	8.27	9.74	7.60	9.04	9.04	8.57	8.28	10.03	7.02	7.49
5	12.06	11.87	10.39	10.02	8.14	6.52	6.52	9.48	9.94	7.30	9.86	10.27
6	7.54	6.55	7.81	7.94	7.81	6.11	6.11	10.63	7.18	7.60	8.23	9.72
7	9.28	8.01	6.49	8.80	8.33	7.38	7.38	7.47	8.04	10.41	10.27	9.63
8	9.90	6.25	8.15	9.72	7.95	7.38	7.38	6.98	9.61	9.03	8.56	7.71
9	8.80	10.30	9.59	8.68	10.68	8.52	8.52	7.56	6.80	9.05	8.91	9.51
10	7.30	8.93	7.37	8.15	7.00	8.54	8.54	7.90	7.57	10.50	8.71	8.93
11	8.18		7.06	7.62	9.12	8.42	8.42	6.99	8.47	10.33	8.58	9.11
12				7.29	7.03	8.27	8.27	6.61		10.30	7.39	6.92

Table 6 continued

Compartment:	Alder Treatment 1	2	3	4	5	6	Hay Treatment 1	2	3	4	5	6
	Dry weight at day 14 (mg)											
1	2.90	3.20	6.70	3.40	4.50	3.10	1.60	1.40	1.90	2.70	1.40	1.30
2	2.40	1.40	3.80	1.40	1.50	2.40	1.60	2.80	3.00	2.60	2.40	1.90
3	1.90	6.00	3.50	1.40	4.50	1.70	2.00	2.60	3.80	3.30	1.50	3.60
4	1.50	1.80	1.40	1.90	2.70	1.70	1.40	2.20	1.60	2.30	3.50	4.10
5	1.10	0.70	3.40	1.30	1.80	1.00	2.00	2.00	1.80		1.90	1.60
6	2.00	0.90	4.50	2.10	1.60	2.10	2.80	4.90	2.10		2.50	1.70
7	1.90	1.40	4.30	1.10	1.80	1.60		2.40	1.60		1.20	2.40
8	2.00	0.80	1.90	1.70	1.20	0.40			2.70		2.40	1.40
9	4.00		2.90	1.10	1.70				2.50		2.30	
10	2.10		2.40		2.70				1.40		3.00	
11	3.10		3.20						1.30		1.00	
12	3.90		2.50						1.40		2.40	
13	1.60		1.10						3.50			
14									1.70			
15									3.50			
16									2.50			
17									1.20			
18									4.90			

Table 6: continued

Compartment:	Alder Treatment 1	2	3	4	5	6	Hay Treatment 1	2	3	4	5	6
	Length at day 14 (mm)											
1	9.35	9.46	12.31	10.24	11.21	10.21	7.51	7.59	7.76	9.28	6.42	6.54
2	11.46	7.80	10.09	7.33	6.81	9.31	7.31	8.93	9.00	8.15	8.84	7.28
3	7.81	11.59	10.62	8.02	10.10	7.03	7.16	9.44	9.39	9.12	6.99	10.01
4	6.85	7.34	6.65	7.70	8.76	7.60	6.31	8.32	7.93	8.09	9.84	10.18
5	6.97	5.84	10.56	7.62	6.98	8.01	8.40	8.91	7.59		7.33	9.73
6	8.99	6.66	10.29	8.46	7.74	10.92	8.72	11.36	7.44		7.70	7.21
7	8.17	7.36	10.95	7.33	8.03	7.04		8.37	7.38		7.24	8.54
8	7.80	7.09	7.96	8.15	6.45	6.10			9.60		8.76	6.65
9	10.18		8.87	7.29	6.65				8.64		8.37	
10	8.67		8.40		8.73				6.74		9.51	
11	10.41		9.46						8.47		7.36	
12	11.57		8.11						7.14		9.59	
13	7.88		6.94						9.33			
14									7.32			
15									10.14			
16									8.27			
17									6.98			
18									9.23			
